# Low interim influenza vaccine effectiveness, Australia, 1 May to 24 September 2017

**DOI:** 10.2807/1560-7917.ES.2017.22.43.17-00707

**Published:** 2017-10-26

**Authors:** Sheena G Sullivan, Monique B Chilver, Kylie S Carville, Yi-Mo Deng, Kristina A Grant, Geoff Higgins, Naomi Komadina, Vivian KY Leung, Cara A Minney-Smith, Don Teng, Thomas Tran, Nigel Stocks, James E Fielding

**Affiliations:** 1 WHO Collaborating Centre for Reference and Research on Influenza, Melbourne, Australia; 2 School of Global and Population Health, University of Melbourne, Australia; 3 Discipline of General Practice, University of Adelaide, Australia; 4 Victorian Infectious Diseases Reference Laboratory, Melbourne, Australia; 5 SA Pathology, Adelaide, Australia; 6 PathWest Laboratory Medicine WA, Perth, Australia; 7 School of Biomedical Sciences, Monash University, Melbourne, Australia

**Keywords:** Australia, viral infections, influenza, influenza-like illness - ILI, surveillance, vaccines and immunisation, epidemiology

## Abstract

In 2017, influenza seasonal activity was high in the southern hemisphere. We present interim influenza vaccine effectiveness (VE) estimates from Australia. Adjusted VE was low overall at 33% (95% confidence interval (CI): 17 to 46), 50% (95% CI: 8 to 74) for A(H1)pdm09, 10% (95% CI: -16 to 31) for A(H3) and 57% (95% CI: 41 to 69) for influenza B. For A(H3), VE was poorer for those vaccinated in the current and prior seasons.

The ongoing Australian 2017 influenza season was so far characterised by record-high laboratory-confirmed influenza notifications [1], high consultation rates, high hospitalisation and mortality rates, particularly in New South Wales [2], large numbers of institutional outbreaks [2] and media attention. The southern hemisphere influenza vaccine used in Australia for this season was a quadrivalent formulation comprised of an A/Michigan/45/2015 (H1N1)pdm09-like virus, an A/Hong Kong/4801/2014 (H3N2)-like virus, a B/Brisbane/60/2008-like virus (of the B/Victoria/2/87 lineage) and a B/Phuket/3073/2013-like virus (of the B/Yamagata/16/88 lineage) [3,4]. This same vaccine composition is being used in the upcoming northern hemisphere for the 2017/18 influenza season [5]. Here we report interim influenza vaccine effectiveness estimates for 2017 in Australia, using sentinel surveillance data.

## Data collection

The Australian Sentinel Practices Research Network (ASPREN) and the Victorian Sentinel Practice Influenza Network (VicSPIN) constitute Australia’s two sentinel influenza general practice (GP) networks. VicSPIN operates in the state of Victoria, while ASPREN operates nationally. Both surveillance systems use similar data collection methods [6,7], with the key difference that the VicSPIN surveillance period is limited to weeks 18 to 43 (1 May 1– 29 October), timed to start roughly 2 weeks after vaccination campaigns in mid-April. Briefly, sentinel GPs submit weekly reports of the number of patients seen with influenza-like illness (ILI), defined as fever/history of fever, cough and fatigue, and the total number of patients. Nose/throat swabs are collected from a subset of patients with demographic data, date of ILI onset, vaccination status (self-reported or medical record) and indications for vaccination, such as belonging to an influenza risk group. Swabs are tested by RT-PCR and positive samples are referred to the World Health Organization (WHO) Collaborating Centre for Reference and Research on Influenza in Melbourne, for antigenic characterisation by haemagglutination inhibition assay (HAI) [8] or focus reduction assay (FRA) [9] and genetic sequencing, as described previously [6]. All data were managed and analysed using R version 3.4.1.

## Influenza-like illness activity

**Figure 1:**
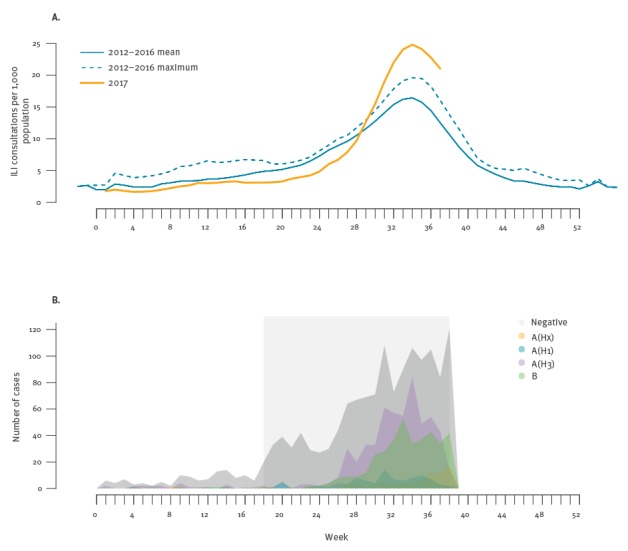
Sentinel general practice (GP) surveillance data^a^, (A) ILI consultation rates, (B) laboratory detections of influenza patients by week and type/subtype, Australia, 2 January–24 September 2017

ILI data from ASPREN for 2017 and 2012 to 2016 (averaged) are plotted in Figure 1A and indicate consultation rates were higher in 2017 than in the previous 5 years. For the study period from 1 May 2017 to 24 September 2017, i.e. weeks 18–38, the 262 ASPREN GPs and 88 VicSPIN GPs conducted 493,961 consultations, of which 5,678 (1.1%) met the ILI case definition and swabs were collected from 2,465 (43%) of them. Influenza cases were detected in every week of the study period and peaked in week 34 (21–27 August, n = 235). Percentage of positive samples peaked in week 32 (7–13 August) with 58%.

ILI: influenza-like-illness.

^a^ Data from the Australian Sentinel Practices Research Network. Victorian Sentinel Practice Influenza Network data (not shown) demonstrated a similar pattern.

In panel A the mean and maximum are calculated by first aligning the peaks for each season, then calculating the weekly mean and maximum consultation rate, as described in [22].

In panel B the shaded rectangular area shows the weeks for which data were included in the vaccine effectiveness analyses, 1 May 2017 to 24 September 2017.

## Virological characteristics

Virological analyses and vaccine effectiveness estimation were restricted to the period 1 May to 24 September (weeks 18-38). During this period, 2,456 patients were swabbed, but samples from 116 patients were excluded because of missing information on vaccination status, and one with missing influenza status. Among the remaining 2,339 patients, working-age adults comprised the majority (n = 1,604, 69%), 440 (19%) were aged < 15 years and 297 (13%) were aged ≥ 65 years. Around 37% (477/1,279) of test-negative patients were vaccinated in 2017.

Eighty-eight patients tested positive for A(H1)pdm09, 522 were A(H3), 75 were not yet subtyped, 11 were B/Victoria, 259 were B/Yamagata and 105 were influenza B, but the lineage was not yet determined (Figure 1B). Virus isolation was attempted for samples with a cycle threshold value of 30 or less (Table 1). HAI testing indicated that isolates were generally antigenically similar to their respective vaccine strains. Thirty-seven percent (n=98) of A(H3) viruses yielded insufficient haemagglutinin titres for testing by HAI and were instead assessed by FRA. In HAI and FRA, 10% (7/67) and 0% (0/75) of A(H3) viruses, respectively, were low reacting to post-infection ferret antisera raised to cell-propagated A/Hong Kong/4801/2014-like viruses. However, these proportions increased to 33% (22/67) and 20% (15/75), respectively, when tested against egg-propagated reference virus.

**Table 1:** Antigenic analysis of virus isolates collected during the influenza season, Australia, 1 May–24 September 2017

Influenza strain	Total viruses attempted^a^	Type of assay	Cell-propagated reference strain		Egg-propagated reference strain^b^	
			Like	Low-reacting	Like	Low-reacting
A(H1)pdm09 A/Michigan/45/2015	55	NA				
Positive	48	HAI	46	0	46	0
Insufficient HA Titre for HAI	1	NA				

A(H3) A/Hong Kong/4801/2014	265	NA				
Positive	90	HAI	60	7	45	22
		FRA	31	0	28	3
Insufficient HA Titre for HAI	98	FRA	44	0	32	12

B/Victoria B/Brisbane/60/2008	7	NA				
Positive	6	HAI	3	1	0	4

B/Yamagata B/Phuket/3073/2013	148	NA				
Positive	90	HAI	52	0	50	2

FRA: focus reduction assay; HAI: haemagglutination inhibition assay. HA: haemagglutinin; NA: not applicable.

^a^ Virus isolation was not attempted for all viruses and not all isolates were analysed by HAI or FRA; FRA was only attempted for A(H3) viruses.

^b^ Results for post-infection ferret antisera raised to egg-propagated and cell-propagated reference viruses are presented because the egg-propagated reference viruses more closely resemble the vaccine strain, while cell-propagated reference viruses more closely resemble the original virus from which the vaccine strain was derived.

**Figure 2:**
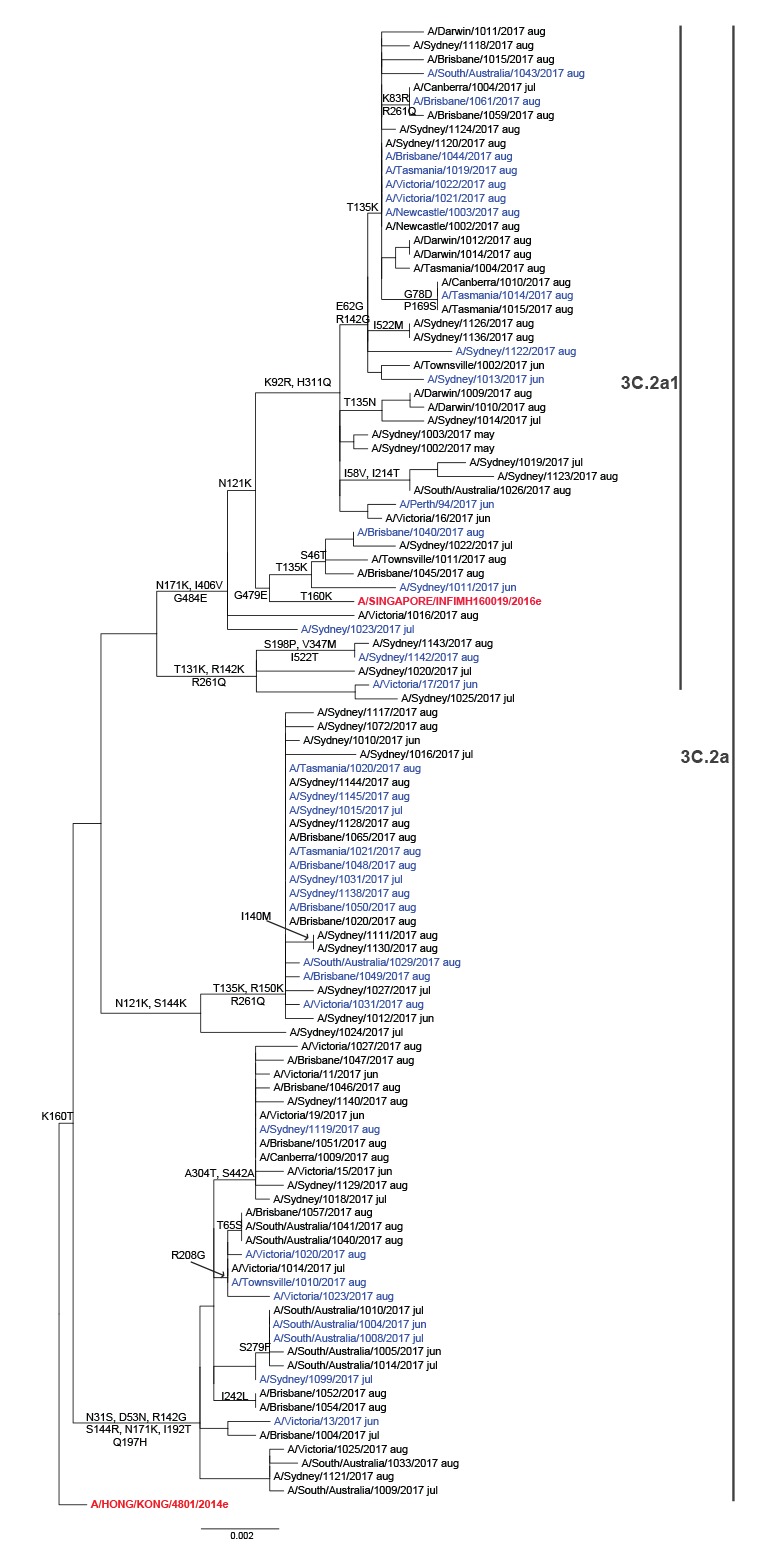
Phylogenetic tree for the haemagglutinin gene of influenza A(H3) viruses

Sequences for the haemagglutinin gene were available for a subset of A(H3) viruses (Figure 2). Examination of the phylogenetic tree identified considerable diversity with a number of viruses exhibiting amino acid substitutions in key glycosylation and antigenic sites but no specific clustering of vaccine failures. GISAID accession numbers for these viruses were 271246, 271303, 275219, 275220, 275225, 275226, 275227, 275228, 275246, 275247, 275248, 275278, 275280, 277305, 277315, 277557.

The phylogenetic tree was created using GENEiOUS, (Biomatters Limited.), Randomized Axelerated Maximum Likelihood (RAxML) Version 8, and Phylogenetic Analysis by Maximum Likelihood (PAML). Blue colouring indicates the virus came from a vaccinated patient. Red colouring indicates vaccine reference viruses. Not all viruses analysed are shown. There were 128 (62%) 3C.2a viruses, among which there were several variants when compared with the vaccine strain, A/Hong Kong/4801/2014, all but three of which included the gain of a potential glycosylation site at position 160 (K160T(+)), which distinguishes these viruses from the vaccine strain. Additional substitutions included 59 viruses characterised by substitutions at N31S,D53N,R142G,S144R,N171K,I192T, 45 by N121K,S144K, and 15 by T131K,R142K,R261Q. Seventy-eight (38%) viruses were 3C.2a1 viruses, among which 13 were characterised by substitutions at N121K,G479E,T135K(-) (loss of a potential glycosylation site at position 135) and 61 by K92R,N121K,H311Q including 34 that also had T135K(-).

## Vaccines effectiveness estimates

Vaccine effectiveness (VE) was estimated following a case–control test-negative design, where VE is estimated from the odds ratio (OR) comparing the odds of vaccination among test-positive and test-negative patients. The limitations of this design have been discussed at length [10,11]. Estimates were adjusted for week of specimen collection (cubic spline with 4 knots), and age group (spline with knots at 5, 15, 35, 65, 75 years).

VE estimates are shown in Table 2. Overall VE was 33% (95%CI: 17 to 46). This estimate appeared to be skewed by the very low estimate for A(H3), which was 10% (95%CI: -16 to 31), whereas estimates were higher for A(H1)pdm09 (VE: 50%; 95%CI: 8 to 74) and B (VE: 57%; 95%CI: 41 to 69). VE for A(H3) 3C.2a viruses was 5% (95%CI: -51 to 40), while the estimate for 3C.2a1 was 19% (95%CI: -42 to 55). For patients vaccinated in the 2016 season, VE for A(H3) was 3–4% regardless of whether they were also vaccinated in 2017. In contrast, the highest VE point estimates for influenza B were observed among those vaccinated in both 2016 and 2017.

**Table 2:** Interim sample characteristics and vaccine effectiveness estimates, Australia, 1 May 2017–24 September 2017

Type/subtype	Age group	Cases				Controls				Adjusted^a^ VE (95% CI)
		Unvaccinated		Vaccinated		Unvaccinated		Vaccinated		
**A or B**	All ages	772	73%	288	27%	802	63%	477	37%	33% (17 to 46)
	Children < 15y	235	94%	14	6%	179	94%	12	6%	16% (-95 to 63)
	Adults 15–64y	512	74%	181	26%	587	65%	322	35%	39% (24 to 51)
	Adults ≥ 65y	25	21%	93	79%	36	20%	143	80%	-3% (-92 to 44)
**A(H1)pdm09**	All ages	74	84%	14	16%	802	63%	477	37%	50% (8 to 74)
	Children < 15y	33	97%	1	3%	179	94%	12	6%	NE
	Adults 15–64y	40	78%	11	22%	587	65%	322	35%	49% (2 to 76)
	Adults ≥ 65y	1	33%	2	67%	36	20%	143	80%	NE
**A(H3)**	All ages	347	66%	175	34%	802	63%	477	37%	10% (-16 to 31)
	Children < 15y	100	94%	6	6%	179	94%	12	6%	17% (-132 to73)
	Adults 15–64y	233	68%	110	32%	587	65%	322	35%	16% (-11 to 36)
	Adults ≥ 65y	14	19%	59	81%	36	20%	143	80%	-20% (-160 to 42)
**A(H3) Clade 3C.2a**	All ages	88	69%	40	31%	802	63%	477	37%	5% (-51 to 40)
	Children < 15y	31	94%	2	6%	179	94%	12	6%	NE
	Adults 15–64y	56	68%	26	32%	587	65%	322	35%	14% (-40 to 49)
	Adults ≥ 65y	1	8%	12	92%	36	20%	143	80%	NE
**A(H3) Clade 3C.2a1**	All ages	52	67%	26	33%	802	63%	477	37%	19% (-42 to 55)
	Children < 15y	14	93%	1	7%	179	94%	12	6%	NE
	Adults 15–64y	37	76%	12	24%	587	65%	322	35%	40% (-14 to 71)
	Adults ≥ 65y	1	7%	13	93%	36	20%	143	80%	NE
**B**	All ages	306	82%	69	18%	802	63%	477	37%	57% (41 to 69)
	Children < 15y	91	96%	4	4%	179	94%	12	6%	NE
	Adults 15–64y	208	82%	45	18%	587	65%	322	35%	63% (48 to 74)
	Adults ≥ 65y	7	26%	20	74%	36	20%	143	80%	10% (-156 to 65)
**B/Victoria**	All ages	11	100%	0	0%	802	63%	477	37%	NE
	Children < 15y	4	100%	0	0%	179	94%	12	6%	NE
	Adults 15–64y	7	100%	0	0%	587	65%	322	35%	NE
	Adults ≥ 65y	0	NA	0	NA	36	20%	143	80%	NE
**B/Yamagata**	All ages	206	80%	53	20%	802	63%	477	37%	45% (22 to 62)
	Children < 15y	71	96%	3	4%	179	94%	12	6%	NE
	Adults 15–64y	130	77%	38	23%	587	65%	322	35%	49% (26 to 66)
	Adults ≥ 65y	5	29%	12	71%	36	20%	143	80%	27% (-162 to 77)
**Repeated vaccination – Influenza A(H1)pdm09**										
Neither season		63	73%	NA	NA	647	52%	NA	NA	Ref
Both 2016 and 2017 seasons		NA	NA	13	15%	NA	NA	395	32%	NE
2017 only		NA	NA	1	1%	NA	NA	59	5%	NE
2016 only		NA	NA	9	10%	NA	NA	132	11%	NE
**Repeated vaccination - Influenza A(H3)**										
Neither season		294	17%	NA	NA	647	52%	NA	NA	Ref
Both 2016 and 2017 seasons		NA	NA	155	30%	NA	NA	395	32%	3% (-29 to 27)
2017 only		NA	NA	14	3%	NA	NA	59	5%	43% (-1 to 71)
2016 only		NA	NA	47	9%	NA	NA	132	11%	4% (-40 to 36)
**Repeated vaccination - Influenza B**										
Neither season		262	72%	NA	NA	647	52%	NA	NA	Ref
Both 2016 and 2017 seasons		NA	NA	57	16%	NA	NA	395	32%	59% (42 to 72)
2017 only		NA	NA	11	3%	NA	NA	59	5%	50% (4 to 76)
2016 only		NA	NA	36	10%	NA	NA	132	11%	22% (-18 to 47)

CI: confidence interval; NA: not applicable; NE: not estimated (because cell counts are fewer than five); Ref: reference category VE: vaccine effectiveness; y: years.

Numbers are shown for all groups regardless of whether VE estimation was attempted. VE was only estimated where cell counts were at least five. Although presented, estimates for children and those aged ≥65 years typically have wide CIs and should be interpreted with caution. Note that the same control group is used for all comparisons within an age group.

^a^ All estimates adjusted for calendar time (cubic spline function with 4 knots). Estimates for all ages were also adjusted for age (cubic spline with knots at 5, 15, 35, 65, 75).

## Discussion

Our interim analysis suggests moderate VE against influenza A(H1)pdm09 and influenza B. However, VE was low against influenza A(H3). The antigenic data reflect ongoing issues with A(H3) candidate vaccine viruses which, when propagated in eggs, rapidly acquire adaptive changes in the haemagglutinin which alter antigenicity. Cell-based vaccines, which are less affected by this, are only licensed in the United States, were not available in Australia in 2017 and will also not be available for the upcoming European season. The significant genetic diversity of circulating viruses, many of which exhibit amino acid substitutions in key antigenic and glycosylation sites, also makes it difficult to select candidate vaccine viruses with high coverage.

This was the second season for which the A/Hong Kong/4801/2015-containing vaccine was used in Australia [3,12], and campaigns currently underway in the northern hemisphere are also using it for a second time [5,13]. During the 2016/17 northern hemisphere season interim VE estimates ranged from 15% (95%CI: –11 to 35) to 43% (95%CI: 29 to 54) [14-17]. It is unclear whether sequential vaccination will result in lower estimates for 2017/18, but our VE estimates were particularly low for people who received vaccine in 2016 and for older adults, 76% of whom were sequentially vaccinated. This finding is consistent with a modelling study which predicts low VE for sequentially vaccinated persons when the vaccine composition is identical, but the antigenic distance between the vaccine and circulating strains is high [18]. However, confounding due to prior infection status and negative interference from pre-2016 vaccines could not be controlled for in our analysis, and may have introduced bias.

In contrast to A(H3), VE estimates for influenza B were moderate and the combined effects of vaccination in 2016 and 2017 did not blunt effectiveness for influenza B, even though the composition remained the same. Similarly, VE for the few A(H1)pdm09 cases recruited was moderate, although low for Australia at 50% (95%CI: 8 to 74), where VEs have ranged from 54% to 79% in the past [6,7]. This was the one component of the 2017 vaccine that was updated since 2016, from A/California/7/2009 to A/Michigan/45/2015.

This study provides interim estimates of the 2017 southern hemisphere influenza vaccine in the outpatient setting and may not apply to inpatient settings or severe illness. Interim estimates can reliably predict final season estimates [19], particularly when made after the peak [20], as is the case here. Should the circulating A(H3) influenza viruses predominate in the 2017/18 northern hemisphere influenza season [21], our results suggest that the vaccine may confer limited protection. Health authorities should consider other influenza prevention measures, including antivirals and health promotion messaging, in the event of a severe season and low VE against A(H3).

## References

[1] Australian Government. Department of Health. Australian Influenza Surveillance Report No 10 – week ending 29 September 2017. Canberra: Department of Health; 2017. Available from: http://www.health.gov.au/internet/main/publishing.nsf/Content/ozflu-surveil-no10-17.htm

[2] NSW Government. Health. Influenza Surveillance Report, Week 39: 25 September to 1 October 2017. North Sydney: NSW Government. [Accessed 25 Oct 2017]. Available from: http://www.health.nsw.gov.au/Infectious/Influenza/Publications/2017/weekending-01102017.pdf

[3] World Health Organization (WHO). Recommended composition of influenza virus vaccines for use in the 2017 southern hemisphere influenza season. Geneva: WHO; 29 Sep 2016. Available from: http://www.who.int/influenza/vaccines/virus/recommendations/2017_south/en/

[4] Australian Government. Department of Health. Therapeutic Goods Administration. 2017 seasonal influenza vaccines. Symonston: Therapeutic Goods Administration; 6 Mar 2017. Available from: https://www.tga.gov.au/media-release/2017-seasonal-influenza-vaccines

[5] World Health Organization (WHO). Recommended composition of influenza virus vaccines for use in the 2017-2018 northern hemisphere influenza season. Geneva: WHO; 2017. Available from: http://www.who.int/influenza/vaccines/virus/recommendations/2017_18_north/en/

[6] Fielding JE Levy A Chilver MB Deng YM Regan AK Grant KA Effectiveness of seasonal influenza vaccine in Australia, 2015: An epidemiological, antigenic and phylogenetic assessment. Vaccine. 2016;34(41):4905-12. 10.1016/j.vaccine.2016.08.067 27577556

[7] Sullivan SG Carville KS Chilver M Fielding JE Grant KA Kelly H Pooled influenza vaccine effectiveness estimates for Australia, 2012-2014. Epidemiol Infect. 2016;144(11):2317-28. 10.1017/S0950268816000819 27125368 PMC9150515

[8] Hobson D Curry RL Beare AS Ward-Gardner A The role of serum haemagglutination-inhibiting antibody in protection against challenge infection with influenza A2 and B viruses. J Hyg (Lond). 1972;70(4):767-77. 10.1017/S0022172400022610 4509641 PMC2130285

[9] Lin Y, Gu Y, McCauley JW. Optimization of a Quantitative Micro-neutralization Assay. J Vis Exp. 2016; (118). 10.3791/54897 28060291 PMC5226418

[10] Foppa IM Haber M Ferdinands JM Shay DK The case test-negative design for studies of the effectiveness of influenza vaccine. Vaccine. 2013;31(30):3104-9. 10.1016/j.vaccine.2013.04.026 23624093

[11] Sullivan SG Tchetgen Tchetgen EJ Cowling BJ Theoretical basis of the test-negative study design for assessment of influenza vaccine effectiveness. Am J Epidemiol. 2016;184(5):345-53. 10.1093/aje/kww064 27587721 PMC5013887

[12] World Health Organization (WHO). Recommended composition of influenza virus vaccines for use in the 2016 southern hemisphere influenza season. Geneva: WHO; 24 Sep 2015. Available from: http://www.who.int/influenza/vaccines/virus/recommendations/2016_south/en/ 26454888

[13] World Health Organization (WHO). Recommended composition of influenza virus vaccines for use in the 2016-2017 northern hemisphere influenza season. Geneva: WHO; 25 Feb 2016. Available from: http://www.who.int/influenza/vaccines/virus/recommendations/2016_17_north/en/

[14] Castilla J Navascués A Casado I Díaz-González J Pérez-García A Fernandino L Combined effectiveness of prior and current season influenza vaccination in northern Spain: 2016/17 mid-season analysis. Euro Surveill. 2017;22(7):30465. 10.2807/1560-7917.ES.2017.22.7.30465 28230523 PMC5322189

[15] Flannery B Chung JR Thaker SN Monto AS Martin ET Belongia EA Interim Estimates of 2016-17 Seasonal Influenza Vaccine Effectiveness - United States, February 2017. MMWR Morb Mortal Wkly Rep. 2017;66(6):167-71. 10.15585/mmwr.mm6606a3 28207689 PMC5657861

[16] Kissling E Rondy M I-MOVE/I-MOVE+ study team Early 2016/17 vaccine effectiveness estimates against influenza A(H3N2): I-MOVE multicentre case control studies at primary care and hospital levels in Europe. Euro Surveill. 2017;22(7):30464. 10.2807/1560-7917.ES.2017.22.7.30464 28230524 PMC5322188

[17] Skowronski DM Chambers C Sabaiduc S Dickinson JA Winter AL De Serres G Interim estimates of 2016/17 vaccine effectiveness against influenza A(H3N2), Canada, January 2017. Euro Surveill. 2017;22(6):30460. . Available from: https://doi.org/10.2807/1560-7917.ES.2017.22.6.30460 10.2807/1560-7917.ES.2017.22.6.30460 28205503 PMC5316907

[18] Smith DJ Forrest S Ackley DH Perelson AS Variable efficacy of repeated annual influenza vaccination. Proc Natl Acad Sci USA. 1999;96(24):14001-6. 10.1073/pnas.96.24.14001 10570188 PMC24180

[19] Leung VK Cowling BJ Feng S Sullivan SG Concordance of interim and final estimates of influenza vaccine effectiveness: a systematic review. Euro Surveill. 2016;21(16):30202. 10.2807/1560-7917.ES.2016.21.16.30202 27124573

[20] Sullivan SG Kelly H Late season interim estimates of influenza vaccine effectiveness reliably predict end of season estimates in Victoria, Australia, 2007 to 2012. Euro Surveill. 2013;18(41):20605. 10.2807/1560-7917.ES2013.18.41.20605 24135124

[21] Pan American Health Organization (PAHO). Weekly Influenza Report EW 39. Washington: PAHO; 11 Oct 2017. Available from: http://www.paho.org/hq/index.php?option=com_docman&task=doc_view&Itemid=270&gid=42470&lang=en

[22] World Health Organization (WHO). A manual for estimating disease burden associated with seasonal influenza. Geneva: WHO; 2015. Available from: http://www.who.int/influenza/resources/publications/manual_burden_of_disease/en/

